# Identification of Potential Extractables and Leachables in Cosmetic Plastic Packaging by Microchambers-Thermal Extraction and Pyrolysis-Gas Chromatography-Mass Spectrometry

**DOI:** 10.3390/molecules25092115

**Published:** 2020-04-30

**Authors:** Pauline Murat, Sowmya Harohalli Puttaswamy, Pierre-Jacques Ferret, Sylvie Coslédan, Valérie Simon

**Affiliations:** 1 Chimie analytique et Compatibilité, Pierre Fabre Dermo-Cosmétique, 17 allée Camille Soula, 31320 Vigoulet-Auzil, France; pauline.murat@ensiacet.fr (P.M.); sylvie.cosledan@pierre-fabre.com (S.C.); 2 Laboratoire de Chimie Agro-Industrielle (LCA), Université de Toulouse, INRA, INPT, 31030 Toulouse, France; sowmyahp22@gmail.com; 3 Safety Assessment Department, Pierre Fabre Dermo-Cosmétique, 3 avenue Hubert Curien, 31035 Toulouse Cedex, France; pierre-jacques.ferret@pierre-fabre.com

**Keywords:** volatile and semi-volatile compounds, migration, plastic packaging, emission, thermal extraction, green analytical chemistry

## Abstract

Most container–content interaction studies are carried out through migration tests on end products or simulants involving generally toxic solvents. This study was conducted with the aim of identifying potential leachables from materials used in cosmetic plastic packaging by using two approaches based on solvent-free extraction, i.e., solid-phase microextraction sampling and pyrolyzer/thermal desorption coupled with gas chromatography mass spectrometry. Volatile and semi-volatile intentionally and non-intentionally added substances were detected in seven packaging samples made of polypropylene, polyethylene, and styrene-acrylonitrile copolymer. Thirty-five compounds related to the polymers industry or packaging industry were identified, among them phthalates, alkanes, styrene, and cyanide derivates including degradation products, impurities, additives, plasticizers, and monomers. All except eight belong to the Cramer class I. These thermodesorption techniques are complementary to those used for migration tests.

## 1. Introduction

Plastic materials are widely used in the packaging industry, particularly in the food, pharmaceutical, medical, and cosmetic fields. The polymers most frequently used for these applications are made of polypropylene (PP), polyethylene (PE), polyvinyl chloride (PVC), polyamide (PA), polyethylene terephthalate (PET), and ethylene vinyl alcohol (EVOH). Packaging must first contain the product and provide physical protection against external aggressions (light, microbiological contamination, oxidation, etc.). It also has to provide information on the product, such as brand, instruction, and ingredients. Most of the time, plastics used for packaging contain additives to give them specific properties such as improved softness, flexibility, or resistance [1,2,3]. Additives can be, among others, plasticizers, ultraviolet (UV) absorbers, antioxidants, dyes, or lubricants [4,5]. Because they are not bound to the polymer matrix, these additives can migrate from the container to the content [6,7], resulting in consumer exposure to chemicals that may potentially be a risk for human health.

These molecules are called extractables or leachables, i.e., molecules that can migrate from the container to the content in extreme or normal conditions. Leaching can be migration of additives but also of non-intentionally added substances, also called NIAS [8,9]. NIAS can be impurities, degradation products, or environmental contaminants [5,10]. Leaching phenomenon can be controlled through evaluations called container–content interaction (CCI) studies. These studies are carried out to monitor extractables and leachables and represent important challenges for industries.

Trace levels of extractables and leachables in matrices, sometimes relatively complex, is a real challenge for analysts, because the tests need to reach very low limits (ppb levels) of detection and/or quantification. Therefore, gas chromatography-mass spectrometry (GC-MS) is often used in CCI studies, for its sensitivity, in food [6,11,12,13,14,15], pharmaceutical [16,17,18,19], medical [20,21], and cosmetic [7,22,23,24,25] studies. Liquid chromatography is also used, whether it is with a UV, fluorescent, [17,26,27,28] or mass spectrometer detector [28,29,30,31,32].

Such studies can be performed with migration tests on end products [22,31] and/or simulants [20,33,34,35,36] that have been in contact with the packaging material. Simulants are simple matrices used to mimic an end product. For food products, the use of simulants is ruled by the regulation n°10/2011 on plastic materials and articles intended to come into contact with food. In this context, six simulants are listed: 10%, 20%, and 50% ethanol, 3% acetic acid, vegetable oil, and poly(2,6-diphenyl-*p*-phenylene oxide) [37]. For cosmetic products, similar simulants, such as ethanol or glycerin, were used by Murat et al. [35,36]. Despite the simplicity of these matrices, they require sample preparation, sometimes fastidiously, and can generate interference in the detection of compounds.

To overcome these problems, CCI studies can/could also be performed on the packaging material itself. The concentration of the unknown migrants could then be much higher, and this could facilitate the identification process. Indeed, thermal extraction combined with pyrolysis (Pyr) or with thermal desorption (TD) associated to GC-MS analysis proved to be a powerful tool to investigate the composition of volatile and semi-volatile compounds. Analytical pyrolysis is mainly used to study the thermal decomposition of synthetic or natural polymers [38,39]. It produces significant amounts of components by cracking and by rearranging fragments depending on the experimental parameters. Thermal desorption is a technique mostly used for air quality studies by extracting volatile compounds sampled on a sorbent prior to GC analysis [40,41], but is also used for the study of volatile organic compounds (VOC) emissions by materials either by inserting them into cartridges [42] or into microchambers (µCTE) [43].

In this context, the aim of this study was to investigate a new approach based on thermal extraction to identify potential leachables from materials used in cosmetic packaging. Two approaches based on solid-phase microextraction (SPME) sampling in microchambers and pyrolysis, both followed by GC-MS analysis are proposed to detect volatile and semi-volatile, intentionally and non-intentionally added substances in cosmetic packaging. Results are compared with those obtained by previous migration studies.

## 2. Results

### 2.1. Identification of Released Compounds

**Figure 1:**
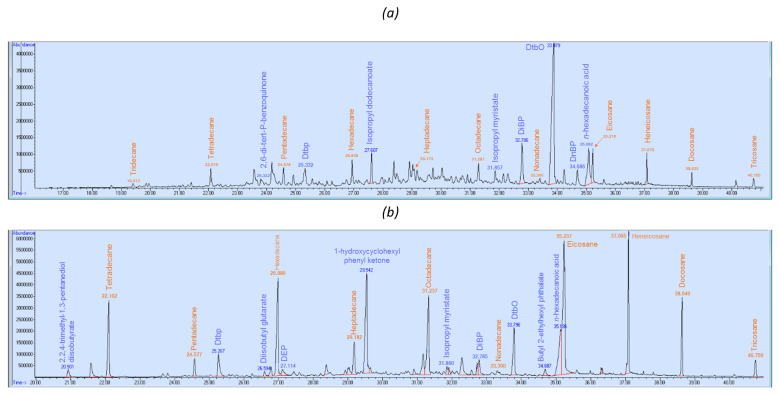
Chromatograms obtained using SPME sampling (PDMS) (T = 110 °C, t_adsorption_ = 30 min) for (**a**) packaging P2 (100% polypropylene (PP)) and (**b**) packaging P6 (coextruded (COEX) 70% linear low density polyethylene (LLDPE)/30% cross-linked low density polyethylene (XLDPE)/ethyl vinyl alcohol (EVOH)).

**Figure 2:**
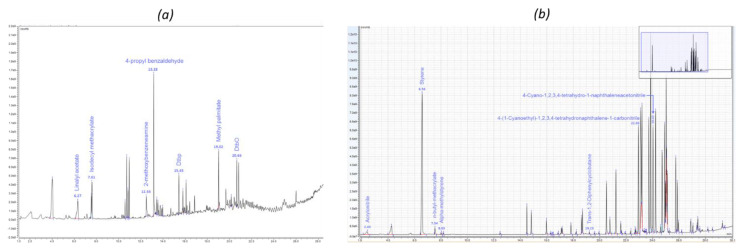
Chromatograms obtained by Pyr-GC-MS for (**a**) packaging P2 (100% PP) and (**b**) packaging P3 (100% styrene-acrylonitrile copolymer (SAN)).

Differences were observed on the obtained chromatograms between the materials, but also between the extraction techniques (Figure 1 and Figure 2). For the SPME-µCTE-GC-MS study, chromatograms obtained at 110 °C vs. at 80 °C showed more intense peaks and were used for the qualitative study. For sample P4, the more abundant peaks were asymmetrical, which modifies the retention time and can also hide other nearby less intense peaks. To overcome this problem, 80 °C chromatograms were also studied.

**Figure 3:**
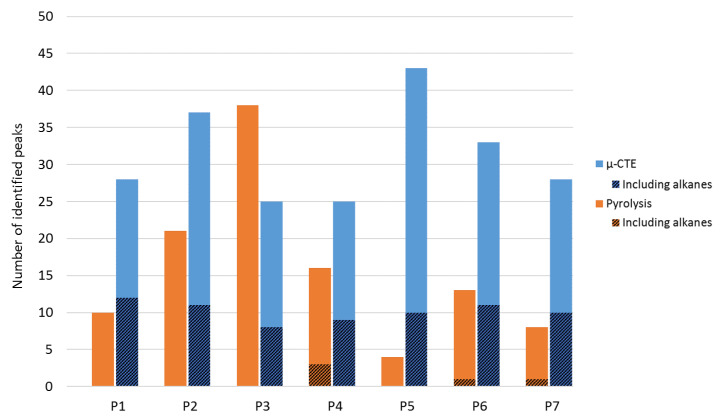
Number of peaks identified in every sample by Pyr (orange) and SPME-µCTE (blue), including alkanes (striped).

An average of 16 identified peaks for pyrolysis and 31 identified peaks for µCTE were identified. Since plastic materials are made up of petroleum products, several peaks were identified as alkanes (Figure 3). Except for P3, more peaks were identified with µCTE than with pyrolysis. This can be explained by the fact that P3 µCTE was realized by bulk emission, while in contrast, the other samples were analyzed by surface emission.

Identified compounds that were related to the polymers industry or packaging industry are listed in Table 1. Only two compounds were detected both with µCTE and pyrolysis: 2,4-di-tert-burylphenol (Dtbp) and 7,9-di-tert-butyl-1-oxaspiro(4,5)deca-6,9-diene-2,8-dione (Dtbo). Moreover, they were detected in almost all the samples analyzed, except Dtbp in P3, which was presented in Groh et al. as one of the known plastic packaging-associated chemicals along with its hazards [44]. Dtbp was also detected as an extractable or leachable in several studies: Jenke et al. found it to be an extractable associated with PP, polyethylene, and polyolefin materials [45], Burman et al. found it in PP films containing Irganox 1010 and Irgafos 168 [46], and Alin et al. [47] found it in PP food packaging. Moreover, it has also been detected in PET and high density PE (HDPE) materials [48,49]. DtbO was also found in PP food packaging [47] and in pouches in PE (internal side) and PET (external side) [50]. In 2011, Löschner proposed a mechanism of degradation of additive Irgafos 168 in Dtbp and of Irganox 1010 in DtbO [51]. Acrylonitrile and styrene were both found by pyrolysis in P3 and can be considered as residual monomers. In the same material, two compounds (4-Cyano-1,2,3,4-tetrahydro-1-naphthaleneacetonitrile and 4-(1-Cyanoethyl)-1,2,3,4-tetrahydronaphthalene-1-carbonitrile) were also detected and are described by Richardson et al. as industrial process undesired by-products [52].

Among the 35 compounds listed, 14 are considered as plasticizers and 8 of these plasticizers are phthalates. Molar masses of the detected molecules were included between 53 (acrylonitrile) and 391 g/mol (diisooctyl phthalate and bis(2-ethylhexyl) phthalate). Furthermore, boiling points varied from 77 °C (acrylonitrile) to around 425 °C (DtbO and 4-(1-Cyanoethyl)-1,2,3,4-tetrahydronaphthalene-1-carbonitrile).

### 2.2. Comparison with Simulants Study

A migration study using aqueous and ethanolic simulants on the same packaging was realized by Murat et al. in 2019 [35]. Ten phthalates were part of the targeted compounds. Comparing results, it appears that DnBP, DEHP, DEP, and DiBP of the µCTE study were also detected in simulants of the same packaging in the migration study. In this previous work, these four compounds were detected in all the packaging samples studied here, except DEHP in P6. Measured concentrations varied from 12.4 µg/L for DiBP in P1 to 491.0 for DiBP in P3, both in simulant ethanol 96%. This illustrates the fact that plastic additives (intentionally or non-intentionally added) can migrate from the material into the product.

However, with µCTE, these four compounds were not detected in all the packaging, compared to the simulant study. Moreover, some phthalates found in the migration study such as benzylbutyl phthalate or diisopentyl phthalate were not detected with the pyrolysis or with the µCTE methods. This can be explained by the fact that phthalates are semi-volatile compounds and they are present in the materials at a trace level. The liquid simulants seemed to present a higher power of extraction than did heat under the conditions of the studies.

A second study on simulants was led by Murat et al. in 2020 [36]. For this work, glycerin and liquid paraffin were used to mimic cosmetic products. The same phthalates were targeted. Once again, DEP, DiBP, DnBP, and DEHP were detected in the simulants in several packaging. Higher concentrations of DEP were measured in glycerin and liquid paraffin both in contact with P5. Only one phthalate detected in glycerin was not detected using µCTE: diisopentyl phthalate in P7.

Comparing migration and extraction studies, it appears that they are complementary. Thermal extractions allow for the identification of numerous potential leachables, without using any solvent, during migration studies. There are limitations to both approaches. To detect all the leachables during migration studies, several chromatographic techniques must be used. Thermal extractions can highlight the presence of numerous compounds, but their capacities to migrate into simulants or products must be verified.

Eventually, thermal extractions can be used to list the additives, NIAS, and all potential leachables present in a material. Then, the migration approach can be used to check which compounds are able to migrate.

### 2.3. Toxicological Aspects

The Cramer class of the compounds presented in the Table 1 were determined using the Toxtree online tool. Cramer class I corresponds to substances with a simple structure and low toxicity. The threshold of toxicological concern for these substances is 1800 µg/person/day. Cramer class II compounds are of medium toxicity and their structures are less inoffensive than structures of class I compounds. Their threshold is 540 µg/person/day. Finally, Cramer class III compounds have reactive functional groups and they present a high toxicity. Their threshold is fixed at 90 µg/person/day.

**Figure 4:**
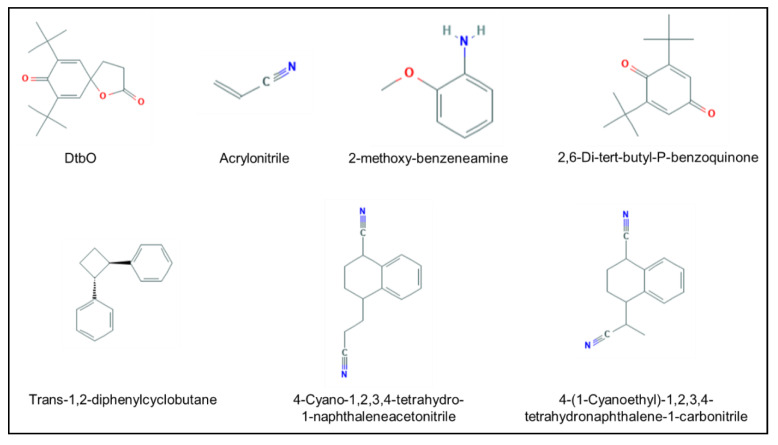
Structures of the detected compounds belonging to the Cramer class III.

**Figure 5:**
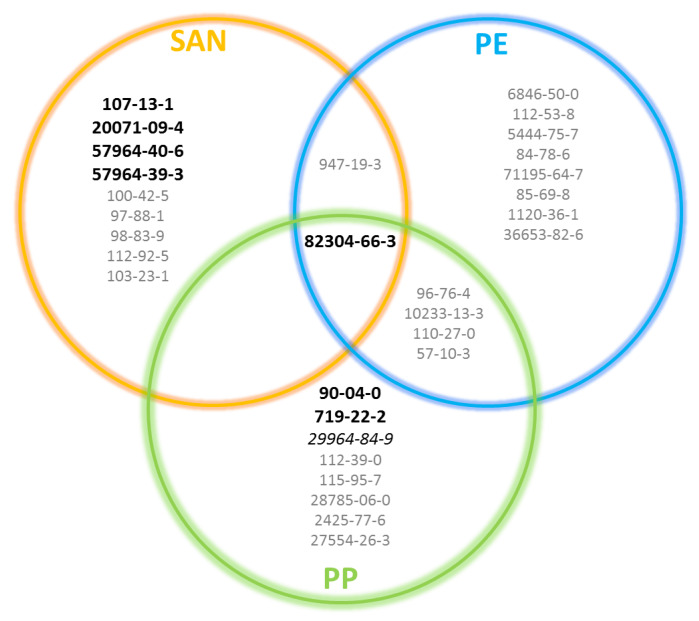
Cartography of the identified compounds to study by packaging materials. Cramer class III compounds are in black and bold, Cramer class II compounds in black and italic, and Cramer class I compounds in grey.

Among the 35 compounds, 27 belong to class I, 1 to class II, and 7 to class III. These last ones are presented in Figure 4. Compounds detected in this work are present in the packaging materials but their ability to migrate into products such as cosmetic formulas were not tested. It would be interesting to perform simulants studies on them, as presented by Murat et al. for phthalates [24]. GC-MS methods could be developed to qualify and quantitate the compounds presented in Figure 5. Priority compounds are ones of the Cramer class III (in bold in the cartography). Finally, with quantitation methods, safety evaluations could be done to ensure that associations the between packaging and products are safe for the consumers.

## 3. Materials and Methods

### 3.1. Samples

Seven packaging options used for cosmetic products were studied. They were all made up of thermoplastics. Two were of polypropylene but from different suppliers in order to evaluate the supplier influence on the leachable profile of packaging. One was of styrene-acrylonitrile copolymer (SAN) and the others were of polyethylene (high density polyethylene (HDPE), linear low density polyethylene (LLDPE), and cross-linked low density polyethylene (XLDPE)). The coextruded (COEX) studied packaging also contained ethyl vinyl alcohol (EVOH). They are described in Table 2.

### 3.2. Samples Preparation

Packaging samples were cut using clean scissors or when necessary (for P3) a Dremel model 300 (Dremel Europe, Breda, NL) for pyrolysis analyses. Approximately 1-2 µg of material were inserted into a quartz tube for Pyr-GC-MS analysis. Surface emission testing was applied to all the packaging except P3 for µCTE extraction. To do so, 6.4 cm diameter circular samples were cut from the packaging samples and put on spacers for microchambers. P3 was analyzed by bulk emission testing (around 1 g). All tests were duplicated.

### 3.3. Instrumentation and Conditions

A Micro-Chamber/Thermal Extractor (µCTE) 250-series (Markes International, Llantrisant, UK) with four micro-chambers (36 mm deep, 64 mm in diameter) was used to heat samples at 80 °C and 110 °C. Emissions were extracted by SPME. A preliminary study was completed concerning material P5 and involving four types of fibers (Supelco, Bellefonte, PA, USA): polydimethylsiloxane (PDMS) 100 µm, carboxen/PDMS 75 μm, divinylbenzene/carboxen/PDMS fiber 50/30 μm, and polyacrylate 85 μm. The chromatographic patterns obtained for the first three fibers are quite similar due to the observed retention times and peak intensity. In contrast, for the polyacrylate fiber, the chromatogram has not only fewer peaks and but also lower intensity. The PDMS fiber makes it possible to detect peaks characterized by a greater retention time amplitude. It was thus chosen for the study. After sample collection, SPME fiber was desorbed for 5 min and analyzed by an Agilent 6890 gas chromatograph coupled with a 5973 Network mass spectrometer. Injection was in splitless mode on a DB5-MS (30 m × 0.25 mm × 0.25 μm) column from Agilent (Les Ulis, France). Helium was used as carrier gas with a flow rate of 1.3 mL/min. The oven temperature was programmed from 50 °C (held 5 min) to 200 °C at 5 °C/min and then to 250 °C at 30 °C/min (held 7 min). Temperatures of the injector, quadrupole, and ion source were 250 °C, 150 °C, and 230 °C, respectively. The MS detector was run in electron impact mode with electron energy of 70 eV. Data acquisition was made in SCAN mode from 35 to 500 amu. The analytical system was controlled by MSD Chemstation software (Agilent, Les Ulis, France) and comprised a library of mass spectra (NIST^®^ Version 2.2).

A CDS 6150 pyrolyzer (Oxford, PA, USA) was used for pyrolysis analyses. Samples were heated at 340 °C for 30 s and the transfer line temperature was 280 °C. The analytical system consisted of a Thermo Scientific Trace 1310 gas chromatograph coupled with a TSQ 9000 mass detector (San José, CA, USA). A ZB-50 capillary column (30 m length, 0.25 mm, 0.25 µm film thickness) was used for separation (Phenomenex, Torrance, CA, USA). Carrier gas was helium at 1.3 mL/min. Injection was in split mode with a ratio of 70:1 to avoid the detector contamination with polymers fragments. The temperature gradient began at 40 °C for 1 min and was then raised to 320 °C at 10 °C/min (held for 10 min). The MS detector was run in electron impact mode with electron energy of 70 eV. Data acquisition was made in SCAN mode from 45 to 600 amu. The analytical system was controlled by Chromeleon^TM^ software (Thermo Fisher Scientific, San José, CA, USA) and comprised a library spectra (NIST^®^ Version 2.2).

### 3.4. Qualitative Analysis

For both techniques, blanks were performed to check that no eventual contamination occurred during the extraction and analytical processes (empty microchamber and empty pyrolysis tube).

Identification was carried out by using the NIST Mass spectral libraries. Only hypotheses of identification with match and reverse match factors above 700 were considered as acceptable.

## 4. Conclusions

SPME-µCTE-GC-MS and Pyr-GC-MS methods allowed for the study of a wide spectrum of compounds without the use of solvents. The studied compounds included phthalates, styrene, and cyanide derivates along with degradation products, impurities, additives, plasticizers, and monomers. Unlike most methods used for migration studies, thermodesorption does not need toxic solvents. This solvent-free technique is compatible with the principles of Green Chemistry. It is an innovative approach to study the container–content interactions in cosmetics.

## Figures and Tables

**Table 1:** NIST^®^ identification of compounds extracted by Pyr and µCTE.

Compound	Molecular Formula	CAS#	Function	Cramer Class	P1	P2	P3	P4	P5	P6	P7
Acrylonitrile	C_3_H_3_N	107-13-1	Intermediate in the synthesis of antioxidants and dyes, monomer [53,54]	III			Pyr				
2-methoxy-benzeneamine	C_7_H_9_NO	90-04-0	Used for dyes manufacturing, printing ink [55]	III		Pyr					
Styrene	C_8_H_8_	100-42-5	Monomer, intermediate [54,56]	I			Pyr				
*n*-butyl-methacrylate	C_8_H_14_O_2_	97-88-1	Monomer, additive [54]	I			Pyr				
Alpha-methylstyrene	C_9_H_10_	98-83-9	Monomer, additive [54]	I			Pyr				
4-propyl-benzaldehyde	C_10_H_12_O	28785-06-0	Additive degradation product [57]	I		Pyr					
Diethyl phthalate (DEP)	C_12_H_14_O_4_	84-66-2	Solvent, plasticizer, extractable associated with polyethylene and PET [45,58]	I			TD	TD	TD	TD	
Linalyl acetate	C_12_H_20_O_2_	115-95-7	Used for plastics manufacturing, lubricant, and additives [59]	I		Pyr					
1-dodecanol	C_12_H_26_O	112-53-8	Plasticizer, lubricant [59]	I					TD		
1-Hydroxycyclohexyl phenyl ketone	C_13_H_16_O_2_	947-19-3	Photo-initiator [58]	I			TD	TD	TD	TD	TD
Diisobutyl glutarate	C_13_H_24_O_4_	71195-64-7	Plasticizer [60]	I						TD	
4-(1-Cyanoethyl)-1,2,3,4-tetrahydronaphthalene-1-carbonitrile	C_14_H_14_N_2_	57964-39-3	By-product of SAN production process [52]	III			PyrTD				
4-Cyano-1,2,3,4-tetrahydro-1-naphthaleneacetonitrile	C_14_H_14_N_2_	57964-40-6	By-product of SAN production process [52]	III			PyrTD				
2,6-di-tert-butyl-P-benzoquinone	C_14_H_20_O_2_	719-22-2	Degradation product, extractable associated with polyethylene materials [45,61]	III		TD					
2,4-di-tert-butylphenol (Dtbp)	C_14_H_22_O	96-76-4	UV stabilizer, antioxidant, degradation product [58,61]	I	Pyr	PyrTD		PyrTD	PyrTD	PyrTD	PyrTD
Isodecyl methacrylate	C_14_H_26_O_2_	29964-84-9	Monomer [54]	II		Pyr					
1-tetradecene	C_14_H_28_	1120-36-1	Monomer, additive [54]	I							TD
2-ethylhexyl benzoate	C_15_H_22_O_2_	5444-75-7	Plasticizer [62]	I					TD		
Isopropyl dodecanoate	C_15_H_30_O_2_	10233-13-3	Additive [54]	I		TD		TD	TD		TD
Trans-1,2-Diphenylcyclobutane	C_16_H_16_	20071-09-4	Extractable associated with polystyrene materials [45]	III			Pyr				
Di-*n*-butyl phthalate (DnBP)	C_16_H_22_O_4_	84-74-2	Plasticizer, catalyst, extractable associated with polyethylene, PET, and polystyrene materials [45,56,58]	I		TD					TD
Diisobutyl phthalate (DiBP)	C_16_H_22_O_4_	84-69-5	Plasticizer, present in printing ink, extractable associated with polyethylene materials [45,58,61]	I		TD			TD	TD	
2,2,4-trimethyl-1,3-pentanediol diisobutyrate	C_16_H_30_O_4_	6846-50-0	Plasticizer, monomer [54,58]	I					TD	TD	TD
*n*-hexadecanoic acid	C_16_H_32_O_2_	57-10-3	Slip agent degradant, monomer, extractable associated with polyethylene and PET [45,54,61]	I		TD				TD	
1-decanol-2 hexyl	C_16_H_34_O	2425-77-6	Additive [54]	I	TD						
1-hexadecanol	C_16_H_34_O	36653-82-4	Monomer, additive [54]	I							TD
7,9-di-tert-butyl-1-oxaspiro(4,5)deca-6,9-diene-2,8-dione (DtbO)	C_17_H_24_O_3_	82304-66-3	Degradation product, impurity of Irganox 1076 [58,61]	III	PyrTD	PyrTD	TD	PyrTD	TD	TD	TD
Isopropyl myristate	C_17_H_34_O_2_	110-27-0	Plasticizer, lubricant, [61]	I		TD		TD	TD	TD	
Methyl palmitate	C_17_H_34_O_2_	112-39-0	Intermediate for resins and defoamer in food contact coatings [61]	I		Pyr					
1-octadecanol	C_18_H_38_O	112-92-5	Ink solvent, plasticizer [61]	I			TD				
Butyl 2-ethylhexyl phthalate	C_20_H_30_O_4_	85-69-8	Plasticizer [63]	I						TD	
Butyl octyl phthalate	C_20_H_30_O_4_	84-78-6	Plasticizer [63]	I					TD		
Bis(2-ethylhexyl) adipate	C_22_H_42_O_4_	103-23-1	Plasticizer, extractable associated with PET [45,61]	I			TD				
Bis(2-ethylhexyl) phthalate (DEHP)	C_24_H_38_O_4_	117-81-7	Plasticizer, extractable associated with PET and polystyrene materials [45,58]	I			TD				
Diisooctyl phthalate	C_24_H_38_O_4_	27554-26-3	Plasticizer [64]	I	TD						

**Table 2:** Studied packaging descriptions.

Code	Material	Appearance	Shape and Type
**P1**	100% PP	Opaque, white	Elliptical bottle
**P2**	100% PP	Opaque, green	Cylindrical bottle
**P3**	100% SAN	Opaque, white	Cylindrical bottle
**P4**	100% HDPE	Opaque, white	Cylindrical bottle
**P5**	70% LLDPE/30% XLDPE	Opaque, white	Cylindrical tube
**P6**	COEX 70% LLDPE/30% XLDPE//EVOH	Opaque, white	Cylindrical tube
**P7**	70% HDPE/30% LLDPE	Opaque, white	Cylindrical tube
